# Routine Endoscopy After Acute Sigmoid Diverticulitis: Would a Sigmoidoscopy be Sufficient?

**DOI:** 10.7759/cureus.17648

**Published:** 2021-09-01

**Authors:** Enda Hannan, Tim Harding, William Duggan, Conor Brosnan, Donal Maguire

**Affiliations:** 1 General Surgery, St Michael's Hospital, Dublin, IRL

**Keywords:** sigmoid diverticulitis, colorectal cancer, colonoscopy, sigmoidoscopy, diverticulitis

## Abstract

Background: Current guidelines suggest that patients should undergo colonoscopy after CT confirmed acute diverticulitis to outrule colorectal cancer (CRC). The aim of this study was to determine if flexible sigmoidoscopy (FS) could be a viable alternative to full colonoscopy following acute sigmoid diverticulitis.

Methods: A retrospective study of 271 patients was performed who were diagnosed with acute sigmoid diverticulitis by CT and subsequently underwent full colonoscopy. Medical records, CT reports, endoscopy reports, and histopathological reports were reviewed.

Results: Sigmoid diverticulosis was confirmed on colonoscopy in all patients. No colorectal malignancies were detected. Adenomatous polyps were found in 16 (5.9%) patients, of which three had polyps detected beyond the sigmoid colon. The overall proportion of abnormalities found beyond the sigmoid colon was 1.1% (n=3).

Conclusion: The detection of CRC cancer in patients undergoing full colonoscopy following an episode of acute sigmoid diverticulitis is rare. Despite this, current guidelines still advocate for endoscopy due to the potentially serious consequences of a missed malignancy. However, given that the area of concern in these cases is the sigmoid colon, FS may be a feasible means of outruling malignancy in the absence of red flag features that would necessitate a full colonoscopy. Our results support this approach, with no CRC detected and a polyp detection rate equivalent to that of the general population. This offers numerous advantages to a full colonoscopy for the patient and health service by being a quicker, cheaper, safer procedure without the need for full bowel preparation or IV sedation.

## Introduction

Acute diverticulitis is common in Western countries and its incidence has been steadily increasing over the past century [1]. The risk of developing colonic diverticulosis in industrialized countries is approximately 60%, with up to 25% of those going on to suffer at least one episode of acute diverticulitis in their lifetime [2]. This accounts for more than 300,000 hospital admissions and $3 billion in healthcare cost per annum [2-3]. The gold standard diagnostic test is abdominal CT, with sensitivity and specificity both greater than 95% [4]. In the context of diverticulae, CT findings such as pericolic fat stranding and thickening of the bowel wall are highly suggestive of acute diverticulitis [4]. However, there is some overlap in the radiological features of acute diverticulitis and colorectal cancer (CRC) [4]. For this reason, professional societies such as the American Society of Colon and Rectal Surgeons (ASCRS) and the American College of Gastroenterology (ACG) recommend that patients undergo colonoscopy four to six weeks after an episode of acute diverticulitis to exclude malignancy [5-6]. This remains the case in spite of the now widespread use of higher-quality 64-slice CT scanners which may adequately distinguish acute diverticulitis from CRC [7].

The necessity of colonoscopy following resolution of acute diverticulitis has been long debated. Several studies have demonstrated that the risk of underlying CRC or advanced polyps in patients with uncomplicated diverticulitis is similar to that of the general population, suggesting that follow-up endoscopy is not necessary beyond those recommended for screening or with atypical features [5, 8-9]. Despite this, it remains a standard practice to perform post-resolution colonoscopy and this continues to be advocated by most guidelines, likely due to the potentially catastrophic consequences of missing a diagnosis of malignancy [2].

By far the most common location for acute diverticulitis is the sigmoid colon, representing more than 85% of cases [10]. For this reason, it is possible that a flexible sigmoidoscopy (FS) may be a viable alternative to full colonoscopy following acute uncomplicated sigmoid diverticulitis. This would serve the purpose of endoscopically visualizing the radiological area of concern with a shorter, cheaper procedure that carries less risk of perforation and can be performed without IV sedation or full oral bowel preparation [11]. While many studies have argued against the need for post-diverticulitis colonoscopy after high quality CT, the appropriateness of replacing full colonoscopy with FS as a means of follow-up investigation has not been thoroughly investigated previously [11].

The aim of this study was to retrospectively evaluate the yield of follow-up colonoscopy performed post resolution of acute sigmoid diverticulitis in detecting pathology beyond the sigmoid colon. From this, we set out to evaluate the potential role of FS in place of full colonoscopy to outrule malignancy following acute sigmoid diverticulitis.

## Materials and methods

A retrospective observational cohort study of patients diagnosed with acute sigmoid diverticulitis by CT that were treated by conservative management and subsequently underwent follow-up colonoscopy in a public teaching hospital was performed over a three-year period. Patients diagnosed with diverticulitis during the study period were identified by means of the hospital inpatient enquiry system. A review of patient medical records and electronic radiology reports was then performed to identify those with a CT-confirmed diagnosis of acute sigmoid diverticulitis and to collect baseline demographic details. Patients who did not have the diagnosis made by CT or who had non-sigmoid diverticulitis were excluded from the study. Thus, all patients with a clinical diagnosis of mild diverticulitis, or Hinchey 0 diverticulitis, were excluded. Details collected from medical records and CT reports were as follows: patient age, gender, presenting symptoms, and modified Hinchey classification [4]. Any diagnostic uncertainty documented in the CT report by the reporting radiologist or suspicion of potential colonic abnormality other than sigmoid diverticulitis was also noted.

Following this, the search functionality of EndoradTM, the electronic reporting system for endoscopy in our centre, was used to identify all patients diagnosed with acute sigmoid diverticulitis who had a subsequent post-resolution colonoscopy. It is a standard practice in our institution to advise all patients diagnosed with acute diverticulitis to undergo a colonoscopy approximately six weeks post-discharge to exclude a co-existent colorectal malignant lesion. Those who did not have an adequately prepped complete colonoscopy were excluded from the study. The colonoscopy reports were assessed for the time between the episode of diverticulitis and colonoscopy as well as the presence and location of diverticulae, colitis, polyps, or malignancy. Histopathology reports were reviewed for any polypectomies or biopsies performed.

All patient data were anonymized for the purpose of this study. No identifying information was retained by the authors or included in this article. Data were analyzed using basic descriptive statistics. Ethical approval was granted by the local audit committee.

**Table 1:** Modified Hinchey Classification.

Modified Hinchey Classification	
Hinchey 0	Clinical mild diverticulitis
Hinchey I	Confined pericolic inflammation/phlegmon (IA), confined pericolic abscess (IB)
Hinchey II	Pelvic/intra-abdominal/retroperitoneal abscess
Hinchey III	Generalized purulent peritonitis
Hinchey IV	Generalized fecal peritonitis

## Results

Patient demographics and radiological findings

During the four-year study period, a total of 319 patients were found to have CT-confirmed acute sigmoid diverticulitis. Of these, 271 underwent a subsequent complete colonoscopy in our center following conservative management of the initial attack and were included in the study. The majority (n=154, 56.8%) of patients were male and the mean age was 49 years (range 32-86 years). In terms of presenting symptoms, 95.2% (n=258) reported left lower abdominal pain, 21.8% (n=59) described acutely altered bowel habit, 6.6% (n=18) had rectal bleeding, and 4.8% (n=13) reported unintentional weight loss. The majority (72.3%, n=196) were classified as Hinchey I diverticulitis. The reporting radiologist documented an inability to exclude underlying colorectal malignancy in 20.7% of cases and recommended endoscopic visualization of the affected area (n=56) (Table 1).

Endoscopic and histopathological findings

The median time lapse between discharge from hospital and follow-up colonoscopy was eight weeks (range 4-20 weeks). Sigmoid diverticulosis was confirmed by colonoscopy in all patients with no sigmoid malignancies detected. No colorectal malignancies outside of the sigmoid colon were detected. Polyps were found in 19 (7%) patients, of which 16 had adenomatous polyps and three had hyperplastic polyps. Of the 16 patients with adenomatous polyps, three had polyps detected beyond the sigmoid colon, with the rest detected in the sigmoid colon and rectum. No patients were diagnosed either macroscopically or microscopically with colitis. The overall proportion of abnormalities found beyond the sigmoid colon was 1.1% (n=3) (Table 2).

**Table 2:** Patient demographics, radiological findings, and endoscopic findings.

Patient demographics (n=271)	
Male-to-female ratio	154:117
Mean age in years	49 (range 32-86)
Clinical presentation	
Left lower abdominal pain	258 (95.2%)
Altered bowel habit	59 (21.8%)
Rectal bleeding	18 (6.6%)
Unintentional weight loss	13 (4.8%)
Radiological Findings	
Inability to outrule malignancy on CT	56 (20.7%)
Hinchey Classification	I: 196 (72.3%), II:71 (26.2%), III: 4 (1.5%), IV: 0 (0%)
Endoscopic Findings	
Sigmoid diverticulosis	271 (100%)
Colorectal malignancy	0 (0%)
Adenomatous polyp	16 (5.9%)
Abnormality beyond the sigmoid	3 (1.1%)

## Discussion

While there is no evidence to suggest that patients with diverticular disease are at an increased risk of CRC, the historically overlapping features of acute sigmoid diverticulitis and sigmoid malignancy on CT scan have previously made the exclusion of CRC on cross-sectional imaging alone difficult [12]. For this reason, endoscopic evaluation of the colon after an episode of acute diverticulitis remains a standard practice [12]. However, the advent of widely available high-resolution CT scanning has improved diagnostic accuracy and is now better able to distinguish the distinct features of these unrelated pathologies [4, 12]. This has challenged the necessity for routine colonoscopy after CT-diagnosed acute diverticulitis in the modern era, with numerous studies reporting that the diagnostic yield for CRC and adenomatous polyps to be no higher than that of the general population undergoing screening, as was the case in the results of the current study [12-14]. Despite this, the current ASCRS, ACG, and Association of Coloproctology of Great Britain and Ireland guidelines continue to recommend endoscopic evaluation of the colonic lumen following left-sided diverticulitis [5-6]. In all of these guidelines, the justification for recommending a colonoscopy is to exclude an occult malignancy at the radiologically abnormal area, despite the fact that the area of concern may be adequately assessed by FS as opposed to a full colonoscopy to the cecum [5-6].

While the potentially devastating outcome of missing an underlying malignancy may be what has caused hesitancy to change practice regarding the utilisation of endoscopy following acute sigmoid diverticulitis, colonoscopy as a procedure is not without risk. One in 2500 colonoscopies result in perforation and 1 in 2000 patients suffer bleeding requiring intervention [15]. Diverticular disease is a recognized risk factor for colonoscopic perforation, meaning this patient’s demographic is at particular risk of complication from a procedure which may be difficult to justify based on the available evidence [16]. Diverticular disease is also a risk factor for challenging colonoscopy, reflected by an association with poor completion rates, significant patient discomfort, and prolonged procedure duration [16]. Colonoscopy is costly for the health service, with an estimated cost of €530 per procedure, and requires approximately 30 min per procedure [15]. The bowel preparation necessary for a full colonoscopy can result in significant morbidity and potentially mortality as a result of renal impairment and electrolyte imbalance, and adverse events as a result of sedation and opioid-based analgesia are well recognized [11]. These factors raise questions regarding the appropriateness of a full colonoscopy as means of endoscopic evaluation following acute sigmoid diverticulitis.

A potential alternative to full colonoscopy for post-resolution assessment of acute sigmoid diverticulitis is FS. Given that the area of concern is the sigmoid colon, this would serve the purpose of endoscopically excluding malignancy at this location while also having a variety of advantages compared to full colonoscopy. FS is a shorter investigation, with typically a 15 min slot or ‘one point’ assigned per procedure according to the Joint Advisory Group on Gastrointestinal Endoscopy, and thus would save time and reduce burden on the health service [17]. This is an important factor considering that the number of patients awaiting routine colonoscopy has increased by 60% in the last 12 months alone, with the potentially devastating impact that this may have on delay in diagnosis and treatment of time-sensitive conditions [18]. FS is also a safer procedure, with the perforation rate of full colonoscopy being two to four times greater than that of FS [11, 16]. FS can also be performed without sedation and does not require full oral mechanical bowel preparation, instead using rectal enemas to provide satisfactory views of the left colon [16]. The use of FS may also be beneficial financially, with the cost for the health service being less than half of that for full colonoscopy [16]. For these reasons, FS may offer a safer, quicker, and cheaper alternative to full colonoscopy that is less burdensome and more comfortable for the patient while still serving the purpose of outruling malignancy at the radiologically abnormal area.

Such a strategy should be considered on a case-by-case basis. It is important to note that, in the presence of ‘red flag’ features of lower gastrointestinal malignancy, such as unexplained iron-deficiency anemia, change in bowel habit to looser stools, or increased frequency or rectal bleeding without anal symptoms, referral for urgent full colonoscopy should still be sought [19]. Age is also an important consideration, and a full colonoscopy should still be considered an essential first line examination for older patients with new onset lower gastrointestinal symptoms [19]. However, in the case of CT-confirmed acute sigmoid diverticulitis that has presented with the classical clinical picture of left iliac fossa pain with the absence of suspicious clinical features, a FS may be a viable alternative to full colonoscopy [16]. Our results highlight the safety and feasibility of such an approach. This study is not without limitations. It is retrospective in nature, taking place in a single center with a relatively modest cohort of patients. Nonetheless, our findings are important. In our study, CT was confirmed to be a highly reliable means of diagnosing acute sigmoid diverticulitis, with no underlying colorectal malignancy detected endoscopically in any patient. The rate of abnormalities found beyond the sigmoid colon was low, with an adenomatous polyp rate comparable to that of the general population undergoing screening. This supports the potential safety and validity of FS as an alternative means of outruling underlying malignancy post-resolution of acute sigmoid diverticulitis.

## Conclusions

The detection of CRC cancer in patients undergoing routine full colonoscopy following an episode of acute sigmoid diverticulitis is rare. Despite this, current guidelines still advocate for endoscopic evaluation due to the potentially serious consequences of a missed malignancy. However, given that the area of concern in these cases is a radiologically abnormal sigmoid colon, FS may be a feasible means of outruling malignancy in the absence of red flag features that would necessitate a full colonoscopy. Our results support this approach, with no colorectal malignancy detected and a polyp detection rate equivalent to that of the general population. This offers numerous advantages to a full colonoscopy for both the patient and the health service by being a quicker, cheaper, and safer procedure without the need for full bowel preparation or IV sedation.
